# Monoprotonated species of 2-amino­malonyl difluoride, [C_3_H_4_F_2_NO_2_][H_2_F_3_]

**DOI:** 10.1107/S2053229624012452

**Published:** 2025-01-08

**Authors:** Dirk Hollenwäger, Dominik Leitz, Valentin Bockmair, Andreas J. Kornath

**Affiliations:** a Department Chemie, Ludwig-Maximilians Universität, Butenandtstrasse 5-13 (Haus D), D-81377 München, Germany; J-PARC Center, Japan Atomic Energy Agency, Japan

**Keywords:** crystal structure, 2-amino­malonyl difluoride, aminoacyl fluoride, vibrational spectroscopy, sulfur tetra­fluoride, com­plexone

## Abstract

The monoprotonated species of 2-amino­malonyl difluoride, namely, 1,3-di­fluoro-1,3-dioxopropan-2-aminium di­hy­dro­gen trifluoride, [C_3_H_4_F_2_NO_2_][H_2_F_3_], was synthesized from sulfur tetra­fluoride in anhydrous hy­dro­gen fluoride (aHF) with [NH_4_][C_3_H_5_NO_4_] as the starting material. The solvent was removed and the salt was dissolved in aHF and crystallized. In the solid state, the three-dimensional network is built by medium–strong N—H⋯F hy­dro­gen bonds and C⋯F contacts. This is the first structure determination of an aminoacyl difluoride and the second of an aminoacyl fluoride.

## Abstract 2

The monoprotonated species of 2-amino­malonyl difluoride, [C_3_H_4_F_2_NO_2_][H_2_F_3_], is particularly inter­esting for the protonation in superacidic media, as well as in Lewis acidic media, due to its two acyl fluoride moieties. The crystal structure is the first single-crystal X-ray diffraction measurement of this com­pound or its salts.

## Introduction

The monoprotonated species of 2-aminomalonyl difluoride, as the [C_3_H_4_F_2_NO_2_][H_2_F_3_] salt, is the second characterized salt of an acyl­amino fluoride, after the synthesis of glycinoyl fluoride (Hollenwäger *et al.*, 2024*a* ▸). Glycinoyl fluoride with protection groups was first characterized by NMR spectroscopy in 1999 by Carpino & Mansour (1999 ▸). The direct synthesis of acyl­amino fluorides was investigated by our group in 2024 (Hollenwäger *et al.*, 2024*a* ▸). The solid state of glycinoyl fluoride was characterized by vibrational and NMR spectroscopy, as well as single-crystal X-ray diffraction (Hollenwäger *et al.*, 2024*a* ▸). [C_3_H_4_F_2_NO_2_]^+^ is the first cation of the group of acyl fluorides which contains two acyl fluoride moieties. The direct synthesis with sulfur tetra­fluoride in an­hy­drous hy­dro­gen fluoride (aHF) applies Kollonitsch’s idea of using HF as a solvent to improve the formation of SF_3_^+^ to activate the de­oxy­fluorinating species (Kollonitsch *et al.*, 1975 ▸). The aHF also performs the function of protonating the NH_2_ group to prevent adduct formation of SF_4_ with the lone pair of the nitro­gen (Hollenwäger *et al.*, 2024*a* ▸; Goettel *et al.*, 2012 ▸; Chaudhary *et al.*, 2015 ▸). [C_3_H_4_F_2_NO_2_][H_2_F_3_] is produced with [NH_4_][C_3_H_5_NO_4_] as the starting material and belongs to the group of com­plexones (Hollenwäger *et al.*, 2024*b* ▸; Anderegg *et al.*, 2005 ▸). Due to the high toxicity of fluoride, its application is highly likely to be limited. The salt could be used in the specialized field of fluorine chemistry, as its two acyl fluoride moieties make it highly inter­esting for conversion in superacidic or Lewis acidic media.

## Experimental

### Synthesis and crystallization

[NH_4_][C_3_H_5_NO_4_] (67 mg, 0.893 mmol) was added in an FEP (fluorinated ethyl­ene propyl­ene) tube reactor. An­hy­drous hy­dro­gen fluoride (aHF, 0.75 ml) and sulfur tetra­fluoride (203 mg, 1.87 mmol) were then added at −196 °C, and the mixture homogenized at room tem­per­a­ture. Excess solvent was removed at −78 °C overnight. The product was a colourless solid that was stable at room tem­per­a­ture. The equation of the formation is shown in Scheme 1.
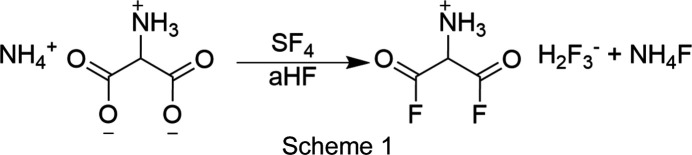


### Analysis (X-ray and Raman)

We investigated and characterized the [C_3_H_4_F_2_NO_2_][H_2_F_3_] salt by single-crystal X-ray diffraction and Raman spectroscopy. Complete data and the devices used for the X-ray measurements are listed in the supporting information (CIF file). Low-tem­per­a­ture Raman spectroscopic studies were performed using a Bruker MultiRAM FT–Raman spectrometer with Nd:YAG laser excitation (λ = 1064 cm^−1^) under vacuum at −196 °C. For measurements, the synthesized com­pounds were transferred to a cooled glass cell.

### Refinement

**Figure 1:**
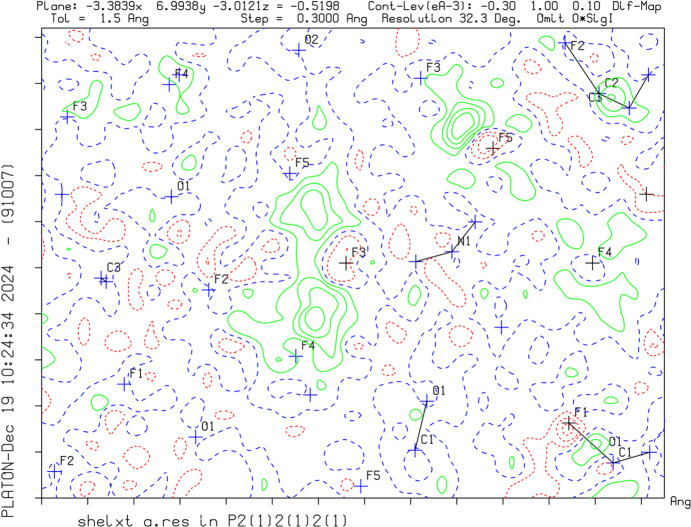
A difference Fourier map of [C_3_H_4_F_2_NO_2_][H_2_F_3_] without the H atoms between F3, F4 and F5, and between N1 and F3, F4 and F5. The green solid lines and red dotted lines show positive and negative density distribution, respectively.

Crystal data, data collection and structure refinement details are summarized in Table 1 ▸. The positions of the H atoms were identified by residual electron-density peaks on the difference Fourier map and by evaluation of the contacts (Fig. 1 ▸). Due to the high diffraction resolution, all H atoms were assigned with respect to the difference map and freely refined.

## Results and discussion

### Single-crystal X-ray diffraction

**Figure 2:**
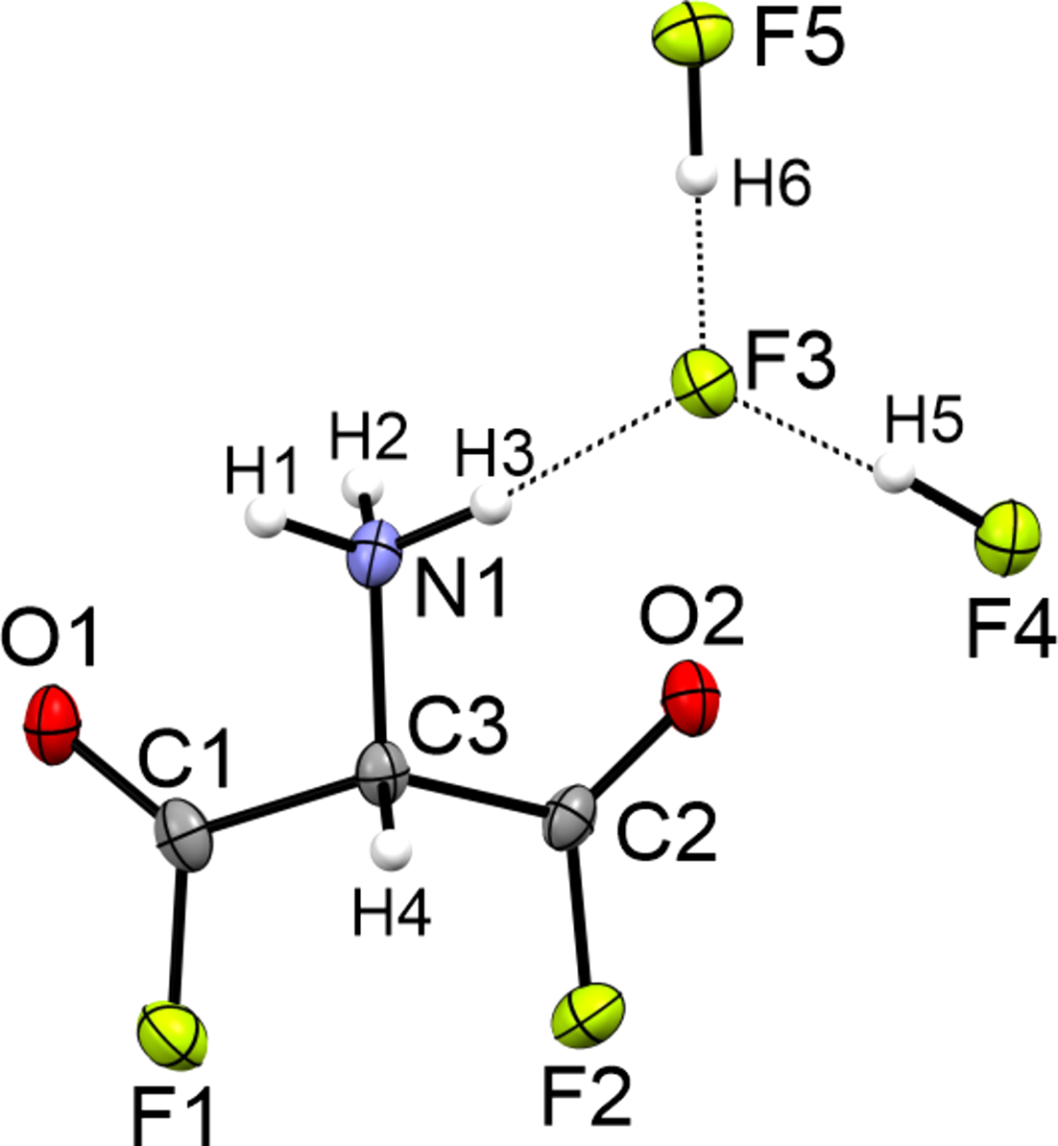
The asymmetric unit of [C_3_H_4_F_2_NO_2_][H_2_F_3_], with displacement ellipsoids drawn at the 50% probability level.

Herein we present the results of the single-crystal X-ray diffraction study of the salt [C_3_H_4_F_2_NO_2_][H_2_F_3_], namely, monoprotonated 2-amino­malonyl difluoride di­hy­dro­gen tri­fluo­ride. The salt crystallizes in the ortho­rhom­bic space group *P*2_1_2_1_2_1_ with four formula units per unit cell. The asymmetric unit is shown in Fig. 2 ▸. The C1—C3 [1.518 (2) Å] and C2—C3 [1.519 (2) Å] bonds are shortened com­pared to those of the starting material [1.539 (2) and 1.549 (2) Å, respectively; Hollenwäger *et al.*, 2024*b* ▸]. The C—C bond lengths are elongated com­pared to the electron diffraction values of malonyl difluoride [1.502 (5) Å; Jin *et al.*, 1992 ▸]. The C1—F1 bond length [1.331 (2) Å] is in the same range as the C2—F2 bond [1.329 (2) Å]. Compared to the C—F bond length of malonyl difluoride (Jin *et al.*, 1992 ▸), the bond in [C_3_H_4_F_2_NO_2_][H_2_F_3_] [1.349 (4) Å] is slightly shortened. The C=O bonds [C1=O1 = 1.175 (2) Å and C1=O2 = 1.172 (2) Å] are in the same range as in malonyl difluoride [1.177 (3) Å; Jin *et al.*, 1992 ▸]. The C3—N1 bond length [1.469 (2) Å] is significantly shortened with respect to the starting material [1.482 (2) Å], the C*sp*^3^—N*sp*^3^ bond lengths (1.488 Å) in organic com­pounds and the bond in glycine [1.482 (3) Å; Hollenwäger *et al.*, 2024*b* ▸; Allen *et al.*, 1987 ▸; Jiang *et al.*, 2015 ▸]. Table 2 ▸ lists the bond lengths, bond angles and torsion angles of the [C_3_H_4_F_2_NO_2_][H_2_F_3_] salt, the starting material [NH_4_][C_3_H_5_NO_4_], glycinoyl fluoride, β-glycine and malonyl di­fluoride (Hollenwäger *et al.*, 2024*a* ▸,*b* ▸; Jiang *et al.*, 2015 ▸; Jin *et al.*, 1992 ▸). The bond deviation of the crystal structure of [C_3_H_4_F_2_NO_2_][H_2_F_3_] with respect to malonyl difluoride results from the structure of malonyl difluoride being measured in the gas phase (Jin *et al.*, 1992 ▸). The shortened C—C bond lengths of the [C_3_H_4_F_2_NO_2_][H_2_F_3_] salt com­pared to the [NH_4_][C_3_H_5_NO_4_] salt is a result of the stronger negative inductive effect of fluorine com­pared to oxygen in the carb­oxy­lic acid. Additionally, the starting material is a zwitterionic anion and the shown aminoacyl fluoride is a cationic salt. The determined structures of glycinoyl fluoride and [C_3_H_4_F_2_NO_2_][H_2_F_3_] are in good agreement with each other (Hollenwäger *et al.*, 2024*a* ▸). The second acyl fluoride moiety of [C_3_H_4_F_2_NO_2_][H_2_F_3_] also exerts a negative inductive effect on the backbone, whereby the C—C bonds are slightly lengthened com­pared to glycinoyl fluoride (Hollenwäger *et al.*, 2024*a* ▸).

The C—C—C angle [114.1 (1)°] is significantly increased com­pared to both the starting material [113.0 (1)°] and malonyl difluoride [110.2 (10)°; Jin *et al.*, 1992 ▸; Hollenwäger *et al.*, 2024*b* ▸]. The C—C—O angles [C3—C1—O1 = 126.0 (2)° and C3—C2—O2 = 125.9 (2)°] are significantly decreased com­pared to malonyl difluoride [129.1 (8)°; Jin *et al.*, 1992 ▸]. The C—C—F angles [111.4 (1) and 111.3 (1)°] are significantly increased com­pared to malonyl difluoride [109.7 (7)°; Jin *et al.*, 1992 ▸]. The C—C—N angles [N1—C3—C1 = 108.5 (1)° and N1—C3—C2 = 108.6 (1)°] are slightly decreased com­pared to the starting material (Hollenwäger *et al.*, 2024*b* ▸). The torsion angles are available in Table 2 ▸.

**Figure 3:**
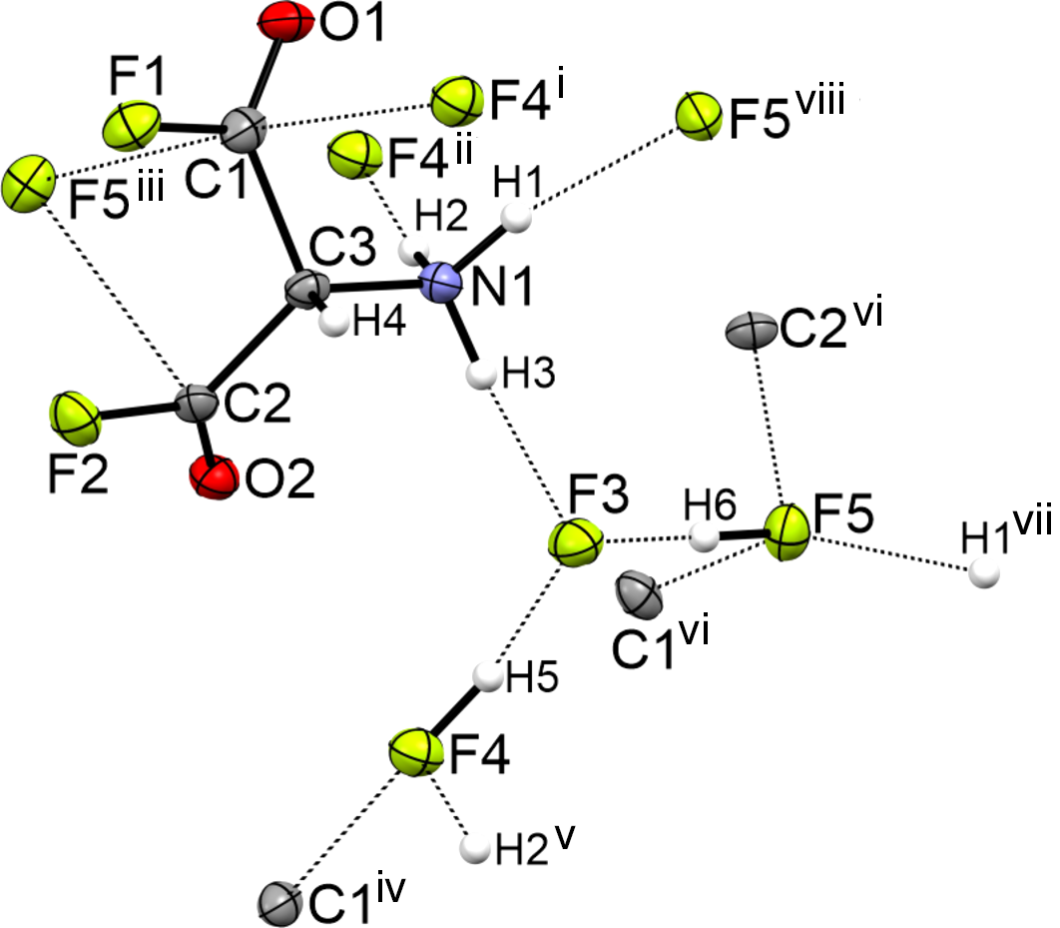
Hydrogen bonds in the crystal structure of [C_3_H_4_F_2_NO_2_][H_2_F_3_], with displacement ellipsoids drawn at the 50% probability level. [Symmetry codes: (i) −*x* + 1, *y* − 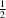, −*z* + 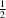; (ii) *x* − 1, *y*, −*z*; (iii) −*x* + 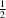, −*y* + 1, *z* − 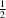; (iv) −*x* + 1, *y* + 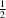, −*z* + 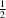; (v) *x* + 1, *y*, *z*; (vi) −*x* + 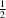, −*y* + 1, *z* + 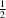; (vii) *x* + 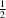, −*y* + 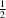, −*z* + 1; (viii) *x* − 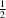, −*y* + 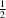, −*z* + 1.]

The crystal structure of [C_3_H_4_F_2_NO_2_][H_2_F_3_] results from a three-dimensional network (Fig. 3 ▸) of two strong hy­dro­gen bonds within the [H_2_F_3_]^−^ anion [2.322 (2) Å for F4(—H5)⋯F3 and 2.338 (2) Å for F5(—H6)⋯F3] and three medium–strong hy­dro­gen bonds classified according to Jeffrey (1997 ▸). The medium–strong hy­dro­gen bonds connect the anion with the cation [2.655 (2) Å for N1(—H3)⋯F3, 2.742 (2) Å for N1(—H1)⋯F5 and 2.743 (2) Å for N1(—H2)⋯F4]. In addition, the network establishes three C⋯F contacts, which are within the van der Waals radii sum (3.17 Å) by approximately 16% [C1⋯F4 = 2.676 (2) Å, C1⋯F5 = 2.682 (2) Å and C2⋯F5 = 2.688 (2) Å]. The hy­dro­gen bonds and C⋯F contacts are listed in the supporting information.

### Raman spectroscopy

**Figure 4:**
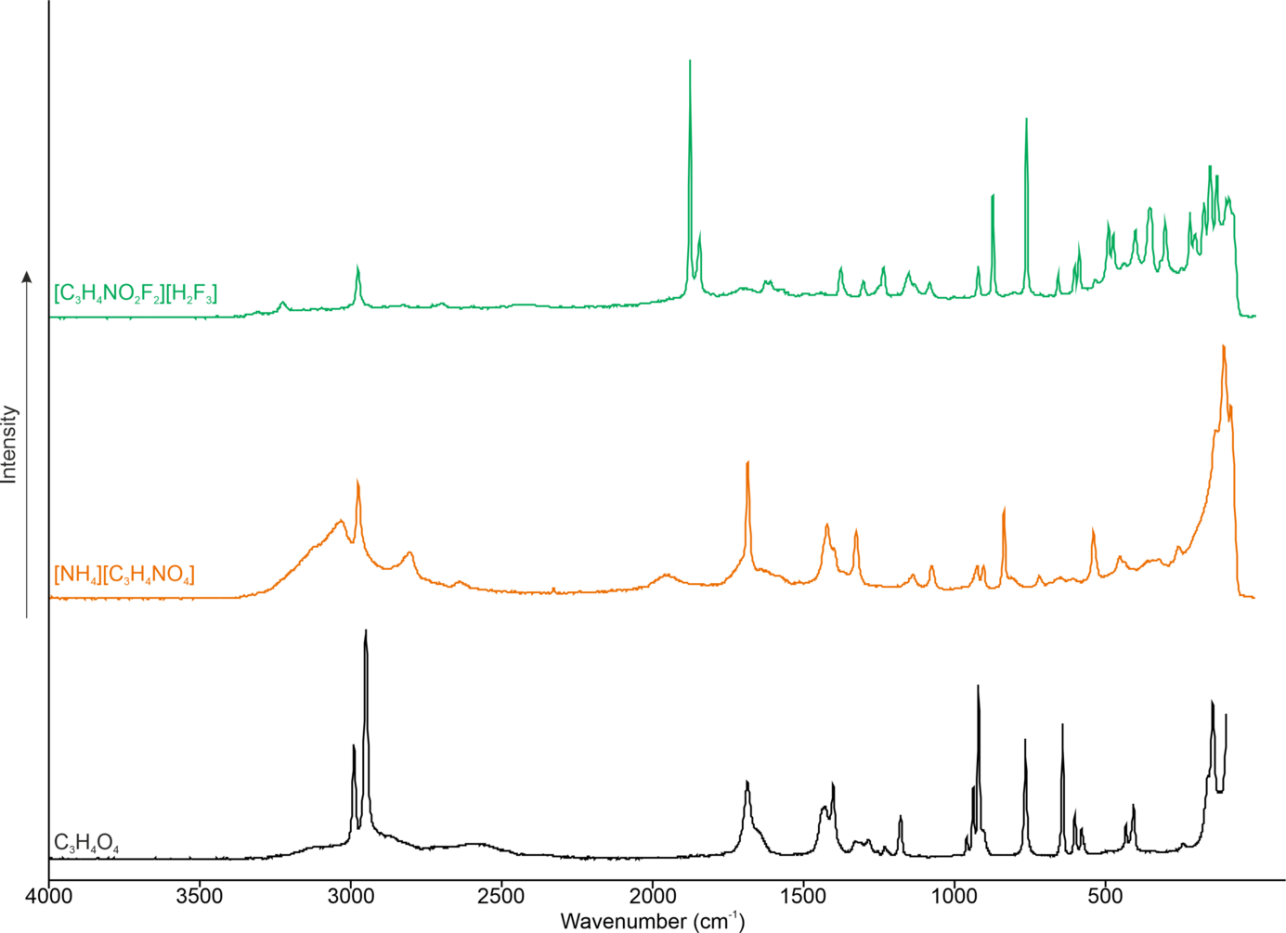
Low-tem­per­a­ture Raman spectra of [C_3_H_4_F_2_NO_2_][H_2_F_3_], [NH_4_][C_3_H_5_NO_4_] and malonic acid (C_3_H_4_O_4_) (Hollenwäger *et al.*, 2024*b* ▸).

The low-tem­per­a­ture Raman spectra of [C_3_H_4_F_2_NO_2_][H_2_F_3_], [NH_4_][C_3_H_5_NO_4_] and C_3_H_4_O_4_ are illustrated in Fig. 4 ▸. Table 3 ▸ lists the Raman data for [C_3_H_4_F_2_NO_2_][H_2_F_3_] and the calculated frequencies for the [C_3_H_4_F_2_NO_2_]^+^ cation at the aug-cc-pVTZ level of theory. The first evidence of the successful preparation of the [C_3_H_4_F_2_NO_2_][H_2_F_3_] salt is the C=O valence oscillation being blue-shifted by approximately 193 cm^−1^ to 1877 and 1846 cm^−1^ com­pared to the starting material at 1684 cm^−1^ (Hollenwäger *et al.*, 2024*b* ▸). The second pieces of evidence are the C—F valence oscillations, which are coupled with the δ(C—H) bands at 1303, 1235 and 1152 cm^−1^. The C—C stretching vibrations are observed at 923 and 763 cm^−1^. Only one of the three N—H vibrations is Raman-active and is observed at 3225 cm^−1^. The C—H stretching oscillation is detected at 2978 cm^−1^.

## Conclusion

Herein we present the first single-crystal X-ray diffraction and Raman spectroscopy study of the [C_3_H_4_F_2_NO_2_][H_2_F_3_] salt. This salt represents the first difluoride in the group of di­acyl­amino fluorides and is a further example of a com­plexone.

## Supplementary Material

Crystal structure: contains datablock(s) I, global. DOI: 10.1107/S2053229624012452/oj3024sup1.cif

Structure factors: contains datablock(s) I. DOI: 10.1107/S2053229624012452/oj3024Isup2.hkl

CCDC reference: 2413368

## Figures and Tables

**Table 1:** Experimental details

Crystal data	
Chemical formula	C_3_H_3_F_2_NO_2_^+^·H_2_F_3_^−^
*M*_r_	183.09
Crystal system, space group	Orthorhombic, *P*2_1_2_1_2_1_
Temperature (K)	102
*a*, *b*, *c* (Å)	5.5736 (2), 9.2154 (4), 12.7952 (7)
*V* (Å^3^)	657.20 (5)
*Z*	4
Radiation type	Mo *K*α
μ (mm^−1^)	0.23
Crystal size (mm)	0.34 × 0.11 × 0.07

Data collection	
Diffractometer	Rigaku Xcalibur Sapphire3
Absorption correction	Multi-scan (*CrysAlis PRO*; Rigaku OD, 2020 ▸)
*T*_min_, *T*_max_	0.945, 1.000
No. of measured, independent and observed [*I* > 2σ(*I*)] reflections	7118, 2187, 1930
*R*_int_	0.034
(sin θ/λ)_max_ (Å^−1^)	0.752

Refinement	
*R*[*F*^2^ > 2σ(*F*^2^)], *wR*(*F*^2^), *S*	0.034, 0.067, 1.02
No. of reflections	2187
No. of parameters	124
H-atom treatment	All H-atom parameters refined
Δρ_max_, Δρ_min_ (e Å^−3^)	0.27, −0.18
Absolute structure	Flack *x* determined using 683 quotients [(*I*^+^) − (*I*^−^)]/[(*I*^+^) + (*I*^−^)] (Parsons *et al.*, 2013 ▸)
Absolute structure parameter	−0.3 (5)

Computer programs: *CrysAlis PRO* (Rigaku OD, 2020 ▸), *SHELXT* (Sheldrick, 2015*a* ▸), *SHELXL2019* (Sheldrick, 2015*b* ▸), *ORTEP-3 for Windows* (Farrugia, 2012 ▸) and *PLATON* (Spek, 2020 ▸).

**Table 2:** Bond lengths (Å), bond angles (°) and torsion angles (°) of [C_3_H_4_F_2_NO_2_][H_2_F_3_], [NH_4_][C_3_H_5_NO_4_], glycinoyl fluoride, β-glycine and malonyl difluoride

Bond length	[C_3_H_4_F_2_NO_2_][H_2_F_3_]	[NH_4_][C_3_H_5_NO_4_]	Glycinoyl fluoride	β-Glycine	Malonyl difluoride
C1—C3	1.518 (2)	1.5394 (18)	1.509 (5)	1.536 (3)	1.502 (5)
C2—C3	1.519 (2)	1.5485 (18)			1.502 (5)
C1—F1	1.331 (2)		1.333 (4)		1.349 (4)
C2—F2	1.329 (2)				1.349 (4)
C1=O1	1.175 (2)	1.2483 (16)		1.257 (2)	1.177 (3)
C2=O2	1.172 (2)	1.2462 (17)		1.258 (3)	1.177 (3)
C3—N1	1.469 (2)	1.4821 (16)	1.476 (5)	1.482 (3)	
					
Angle					
C1—C2—C3	114.1 (1)	113.00 (10)			110.2 (10)
C3—C1—O1	126.0 (2)	117.7 (1)	127.2 (3)		129.1 (8)
C3—C2—O2	125.9 (2)	116.9 (1)			129.1 (8)
C3—C1—F1	111.3 (1)		110.2 (3)		109.7 (7)
C3—C2—F2	111.4 (1)				109.7 (7)
N1—C3—C1	108.5 (1)	109.56 (10)	108.3 (3)	111.8 (1)	
N1—C3—C2	108.6 (1)	109.98 (10)			
					
Torsion angle					
F1—C1—C3—N1	−173.5 (1)				
F1—C1—C3—C2	65.4 (2)				
O1—C1—C3—N1	4.7 (2)	−2.96 (16)	8.1 (5)		
O1—C1—C3—C2	−116.4 (2)	120.0 (1)			112.0 (20)
F2—C2—C3—N1	177.7 (1)		−171.2 (3)		
F2—C2—C3—C1	−61.2				
O2—C2—C3—C1	122.4 (2)	−112.3 (1)			0.0

**Table 3:** Experimental (exptl) Raman vibrational frequencies (cm^−1^) of [C_3_H_4_F_2_NO_2_][H_2_F_3_] and calculated (calc) Raman vibrational frequencies (cm^−1^) of [C_3_H_4_NO_2_F_2_]^+^ at the M062x/aug-cc-pVTZ-level of theory (the scaling factor is 0.956)

Exptl	Calc	Assignment		
	3327 (173)	A	ν1	ν(NH)
3225 (6)	3182 (251)	A	ν2	ν(NH)
	3143 (67)	A	ν3	ν(NH)
2978 (19)	2980 (22)	A	ν4	ν(CH)
1877 (100)	1889 (297)	A	ν5	ν(CO)
1846 (31)	1862 (210)	A	ν6	ν(CO)
1627 (14)	1578 (34)	A	ν7	δ(NH_2_)
1611 (14)	1560 (44)	A	ν8	δ(NH_2_)
1378 (19)	1446 (187)	A	ν9	δ(NH_2_)
1303 (14)	1341 (251)	A	ν10	ν(CF)+δ(CH)
	1255 (98)	A	ν11	ν(CF)+δ(CH)
1235 (20)	1237 (208)	A	ν12	δ(CH)+ν(CF)
1152 (17)	1160 (59)	A	ν13	δ(CH)+ν(CF)
1133 (13)	1092 (43)	A	ν14	δ(CH)+δ(NH_3_)
1083 (14)	1080 (8)	A	ν15	δ(CH)+δ(NH_3_)
	1030 (16)	A	ν16	ν(CN)
923 (20)	895 (51)	A	ν17	ν(CC)
873 (47)	858 (25)	A	ν18	δ(CCC)
763 (77)	755 (1)	A	ν19	ν(CC)
657 (17)	650 (32)	A	ν20	δ(COF)
603 (20)	588 (3)	A	ν21	ρ(CCC)
587 (25)	576 (15)	A	ν22	δ(NH_3_)
533 (15)				
491 (36)				
475 (32)	486 (47)		ν23	δ(NH_3_)
439 (21)				
402 (34)	380 (18)	A	ν24	δ(CCF)
352 (43)	303 (14)	A	ν25	δ(CCF)
248 (20)	287 (14)	A	ν26	δ(NH_3_)
221 (41)				
203 (32)	191 (0)	A	ν27	δ(NH_3_)
155 (59)	160 (11)	A	ν28	δ(COF)
130 (55)				
99 (46)	93 (14)	A	ν29	τ(COF)
90 (47)				
80 (41)	45 (0)	A	ν30	τ(COF)
